# Rising Trends and Safety of Outpatient Robotic Partial Nephrectomy: A Propensity-Matched National Analysis

**DOI:** 10.7759/cureus.105785

**Published:** 2026-03-24

**Authors:** Sri Saran Manivasagam, Blake R Baer, Jay D Raman, Matthew G Kaag

**Affiliations:** 1 Department of Urology, Penn State University College of Medicine, Milton S. Hershey Medical Center, Hershey, USA

**Keywords:** minimally invasive partial nephrectomy, outpatient cancer surgery, outpatient surgery, robotic partial nephrectomy, urologic oncology

## Abstract

Introduction

Minimally invasive partial nephrectomy (MIPN), laparoscopic or robotic partial nephrectomy (RPN), is the preferred treatment for T1 renal cell carcinoma (RCC). As outpatient surgery expands across urology, its safety and feasibility in oncologic procedures, like MIPN and especially RPN, remain underexplored.

Methods

Using American College of Surgeons National Surgical Quality Improvement Program (ACS-NSQIP) data (2022-2023), we identified 8,927 adult patients who underwent RPN. Trends in outpatient MIPN, both laparoscopic and robotic approaches, were observed from 2019 to 2023. Patients who underwent RPN were then categorized by inpatient or outpatient setting. Propensity score matching was applied to balance demographic and clinical variables. Postoperative complications and readmissions were compared, and multivariate logistic regression identified predictors of adverse outcomes such as 30-day infectious complications, reoperations, and readmissions.

Results

Outpatient MIPN increased from 20.8% (n = 884) in 2019 to 35.5% (n = 1827) in 2023. After matching, 3,180 inpatient and 3,185 outpatient cases of RPN were analyzed. With the current patient selection criteria, outpatient RPN was associated with significantly lower rates of pneumonia, pulmonary embolism, myocardial infarction, deep vein thrombosis, septic shock, blood transfusions, reoperation, and readmissions. Logistic regression identified inpatient setting as an independent predictor of infectious complications (odds ratio (OR): 1.31), reoperation (OR: 1.92), and readmission (OR: 1.23). While outpatient surgery was associated with lower complication rates, this likely reflects the selection of healthier patients for ambulatory care rather than an intrinsic advantage of the outpatient setting.

Conclusion

Outpatient MIPN is increasingly utilized, and RPN demonstrates non-inferior safety compared to inpatient procedures in appropriately selected patients. However, disparities in access and limitations in surgical and oncologic data warrant further investigation. These findings support the safe expansion of outpatient RPN, emphasizing the need for standardized protocols, equitable access, and future research into long-term outcomes and individualized risk stratification.

## Introduction

Renal cell carcinoma (RCC) is a common genitourinary malignancy, affecting over 350,000 individuals in 2019 and accounting for more than 150,000 deaths in the same year [1]. Since 2010, there has been a steady increase in newly diagnosed cases of RCC at a rate of 1.1% annually [2]. In the United States, the average one-year per-patient total cost excluding prescription drug costs was nearly three times higher for patients with RCC compared to controls ($57,588 vs. $19,297). Given the increasing incidence of RCC, an aging population, and longer survival translating to prolonged surveillance, the costs associated with RCC management are expected to escalate further [3]. The financial burden on Medicare is also significant, as it provides healthcare coverage to approximately 45% of RCC patients [4].

Surgical excision remains the standard of care for localized RCC, typically performed via radical nephrectomy or partial nephrectomy [5]. For T1 tumors, partial nephrectomy is the preferred treatment due to its nephron-sparing benefits and equivalent oncological outcomes compared to radical nephrectomy [6]. Minimally invasive partial nephrectomy (MIPN) can be performed using laparoscopic or robot-assisted approaches, with robotic partial nephrectomy (RPN) offering advantages such as lower conversion rates, fewer complications, and reduced positive surgical margins [7].

Outpatient surgeries are increasingly common in urology, particularly for benign conditions, and are gradually being adopted for oncologic procedures as well [8]. A study by Madhusoodanan et al. reported that more than 75% of urologic surgeries at an academic medical center were performed on an outpatient basis [9]. However, same-day discharge following partial nephrectomy remains less explored and is a topic of ongoing debate. Johnson et al., analyzing data from the Michigan Urologic Surgery Improvement Collaborative-Kidney database involving 807 patients undergoing minimally invasive renal surgery, found the median length of stay for MIPN to be two days. They proposed that, due to the complexity of partial nephrectomy compared to radical nephrectomy, MIPN should be classified as an inpatient procedure [10].

Given the rising costs of RCC treatment and the increasing emphasis on patient-centered care, outpatient RPN may offer a means to reduce healthcare expenditures while enhancing patient satisfaction. However, the technical complexity of RPN may limit its feasibility in an outpatient setting. Given the conflicting data, the present study aims to evaluate the safety of outpatient RPN compared to inpatient RPN. The primary objective of this study was to compare 30‑day postoperative outcomes (readmissions, reoperations) between outpatient and inpatient RPN cases using propensity‑matched American College of Surgeons National Surgical Quality Improvement Program (ACS-NSQIP) data. The prespecified primary endpoint was 30‑day unplanned readmission. A secondary aim was to describe national trends in outpatient versus inpatient MIPN from 2019 to 2023

## Materials and methods

This retrospective cohort study was conducted using data from the ACS-NSQIP. The ACS-NSQIP collects data on over 150 variables, including preoperative comorbidities, intraoperative variables, and 30-day postoperative morbidity and mortality outcomes for patients undergoing major surgical procedures in both inpatient and outpatient settings. Patients aged 18-90 years who underwent MIPN were identified using the Current Procedural Terminology (CPT) code 50543. The ACS-NSQIP and the participating hospitals are the sources of the data used in this study; however, they have not verified and are not responsible for the statistical validity of the analysis or the conclusions drawn by the authors.

Only patients with complete data were included in the analysis. Patients were categorized into inpatient and outpatient cohorts based on the clinical setting recorded in the database, corroborated by the length of stay. Outpatient surgery was defined as cases classified as outpatient in the database with a length of stay of one day or less, while inpatient surgery included cases classified as inpatient with a length of stay greater than one day. Outpatient surgery was defined as cases classified as outpatient in the ACS‑NSQIP database and corroborated by a length of stay ≤1 day. This definition aligns with NSQIP’s operational distinction between inpatient and outpatient encounters and is widely used in NSQIP‑based surgical outcomes research. Because NSQIP does not reliably differentiate true same‑day discharge (LOS = 0) from overnight observation billed as outpatient care, using LOS ≤1 day minimizes misclassification.

Trends in the performance of MIPN in outpatient and inpatient settings were analyzed from 2019 to 2023. Analysis of RPN was performed only in the years 2022 and 2023, due to the absence of this data in the previous year's databases. Demographic, clinical, and operative characteristics were compared across the cohorts. A rigorous propensity score matching was performed using all demographic, clinical, and operative variables to minimize selection bias. Propensity score matching was performed using 1:1 nearest‑neighbor matching without replacement and a caliper width of 0.2 standard deviations of the logit of the propensity score. The matched cohorts were then compared based on 30-day postoperative outcomes, including surgical site infections, venous thromboembolism, cardiovascular events, blood transfusions, secondary procedures, and readmissions.

In addition, a logistic regression analysis was conducted to evaluate whether the clinical setting (inpatient vs. outpatient) was associated with increased odds of 30-day infectious complications, secondary procedures, and readmissions. Covariates included in the regression model were: age > 75 years, body mass index (BMI) > 30 kg/m², smoking status, dependent functional status, diabetes mellitus (DM), chronic obstructive pulmonary disease (COPD), hypertension, congestive heart failure, chronic kidney disease (CKD), American Society of Anesthesiologists (ASA) Classification 3/4, operation time longer than 180 minutes, and stent placement during surgery along with the clinical setting (outpatient vs. inpatient). All statistical analyses were performed using IBM SPSS Statistics for Windows, version 30.0 (released 2024, IBM Corp., Armonk, NY), and p-values < 0.05 were considered statistically significant.

## Results

Temporal trends in outpatient MIPN from 2019 to 2023

**Figure 1:**
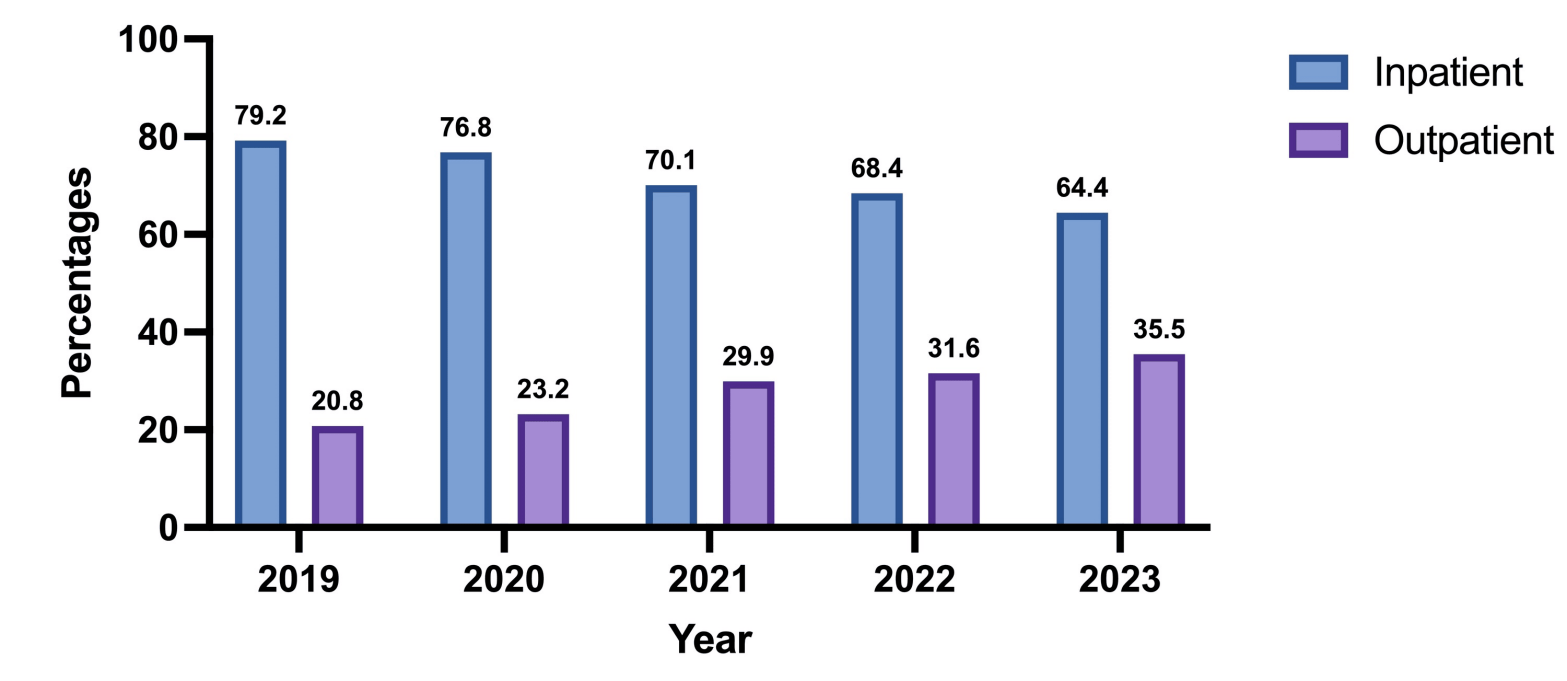
Trends in outpatient and inpatient minimally invasive partial nephrectomy (MIPN) surgery from 2019 to 2023

A total of 22,704 cases that underwent MIPN between 2019 and 2023 were included in the analysis. Out of these, 71.3% (16,193) of cases were performed on an inpatient basis, and 28.6% (6511) were performed on an outpatient basis. Between 2019 and 2023, there has been a steady increase in the proportion of MIPN cases being performed on an outpatient basis, steadily increasing from 20.8% (n = 884) in 2019 to 35.5% (n = 1827) in 2023. This trend reflects a gradual increase in the proportion of cases being performed as ambulatory cases at NSQIP reporting centers (Figure 1).

Baseline characteristics, propensity matching, and 30-day outcomes

Among the 8,927 total cases of RPN from 2022 to 2023, 5,732 cases of inpatient and 3,195 cases of outpatient RPN were included in the analysis. The mean duration of stay in inpatient RPN was 2.17 days (95% CI = 2.13-2.25). Prior to propensity matching, the inpatient and outpatient cohorts differed significantly in terms of race (White race 64.4% (n = 3,694) vs. 75.8% (n = 2,421), p < 0.001), ethnicity (Hispanic ethnicity 12.1% (n = 696) vs. 4.3% (n = 138), p < 0.001), operation time (165.2 vs. 185.8 minutes, p < 0.001), stent placement rates (3.4% (n = 195) vs. 2.3% (n = 73), p < 0.001), prevalence of diabetes (24.3% (n = 1,392) vs. 21.3% (n = 680), p < 0.001), and American Society of Anesthesiologists Grade 3 or 4 (65.2% (n = 3,737) vs. 61.5% (n = 1,965), p < 0.001). The two cohorts did not differ in terms of age, gender, body mass index (BMI), lab values such as serum creatinine (mean 1.01 vs. 1.00 mg/dl, p = 0.15), albumin (4.23 vs. 4.2 gm/dl, p = 0.056), hematocrit (41.9 vs. 42%, p = 0.61), and platelet count (2.50 vs. 2.51 in hundred thousand/mm^3^, p = 0.85), and prevalence of comorbidities such as hypertension (61.8% (n = 3,542) vs. 60% (n = 1,918), p = 0.098), chronic obstructive pulmonary disease (4% (n = 229) vs. 3.3% (n = 107), p = 0.145), heart failure (3.2% (n = 183) vs. 2.6% (n = 82), p = 0.079), and chronic kidney disease requiring dialysis (0.2% (n = 10) vs. 0.2% (n = 6), p = 0.886).

After propensity matching for all the factors mentioned in Table 1, such as age, demographics, comorbidities, and lab values, 3,180 cases of inpatient MIPN and 3,185 cases of outpatient RPN were included in the analysis. Despite matching, the variations of the cohort in terms of race (p = 0.03), ethnicity (p = 0.012), and operation times (p < 0.001) could not be matched. Moreover, the prevalence of diabetes (23% (n = 731) vs. 21.2% (n = 675), p = 0.03) was still significant despite matching, and heart failure (3.1% (n = 98) vs. 1.9% (n = 60), p < 0.001), which was non-significant prior to matching, became significant on matching. After propensity matching, there were no differences in terms of deep surgical site infections (SSIs) (0.06% (n = 2) vs. 0.09% (n = 3), p = 0.929), organ space SSI (0.8% (n = 25) vs. 0.5% (n = 17), p = 0.227), wound disruption (0.09% (n = 3) vs. 0.1% (n = 4), p = 0.402), urinary tract infection (UTI) (1.6% (n = 51) vs. 1.2% (n = 39), p = 0.132), stroke (0.2% (n = 6) vs. 0.1% (n = 2), p = 0.223), sepsis (0.5% (n = 16) vs. 0.3% (n = 11), p = 0.322), and unplanned readmissions related to the procedure (3.7% (n = 118) vs. 2.8% (n = 90), p = 0.095). However, the inpatient cohort had a significantly higher incidence of pneumonia (0.9% (n = 28) vs. 0.4% (n = 16), p = 0.009), pulmonary embolism (0.5% (n = 16) vs. 0.2% (n = 7), p = 0.032), deep venous thrombosis (0.5% (n = 16) vs. 0.3% (n = 6), p = 0.044), myocardial infarction (0.3% (n = 9) vs. 0.1% (n = 2), p = 0.029), septic shock (0.2% (n = 6) vs. 0.01% (n = 1), p = 0.047), reoperation (1.5% (n = 48) vs. 0.8% (n = 24), p < 0.001), and any readmission (4.9% (n = 156) vs. 3.7% (n = 118), p = 0.009) (Table 2).

**Table 1:** Baseline demographic, clinical and laboratory parameters across outpatient and inpatient partial nephrectomy, before and after propensity matching. SD: standard deviation, BMI: body mass index, DM: diabetes mellitus, COPD: chronic obstructive pulmonary disease, HTN: hypertension, ASA: American Society of Anesthesiologists Physical Classification System

	Before propensity matching			After propensity matching		
	Inpatient (n = 5,732)	Outpatient (n = 3,195)	p-value	Inpatient (n = 3,180)	Outpatient (n = 3,185)	p-value
Age in years, Mean, SD	59.3, 12.2	60.4, 12.1	0.20	60.2, 12.4	60.3, 12.4	0.53
Race(n, %)						
White	3694, 64.4%	2421, 75.8%	<0.001	2351, 73.9%	2411, 75.7%	0.03
Black	611, 10.6%	341, 10.7%		353, 11.1%	341, 10.7%	
Asian	244, 4.2%	115, 3.6%		140, 4.4%	115, 3.6%	
Other races	114, 2%	88, 2.7%		110, 3.4%	88, 2.7%	
Unknown	1070, 18.6%	230, 7.2%		226, 7.1%	230, 7.2%	
Ethnicity (n, %)						
Non-Hispanic	4482, 78.2%	2690, 84.1%	<0.001	2664, 83.8%	2684, 84.1%	0.012
Hispanic	696, 12.1%	138, 4.3%		146, 4.6%	137, 4.3%	
Unknown	554, 9.7%	367, 11.1%		362, 11.4%	364, 10.3%	
Gender, (n, %)						
Male	3534, 64.8%	1916, 60%	0.118	1970, 62%	1912, 60.2%	0.208
Female	2198, 38.3%	1279, 40%		1210, 38%	1273, 39.8%	
BMI in kg/m^2^ Mean, SD	32.35, 7.5	32.33, 7.3	0.14	32.3, 7.4	32.3, 7.3	0.11
Operation time, in minutes Mean, SD	165.2, 63.2	185.8, 72.9	<0.001	165.3, 63.2	192.4, 76.7	<0.001
Stent placement during surgery	195, 3.4%	73, 2.3%	<0.001	73, 2.3%	70, 2.2%	0.23
Serum Creatinine, in mg/dl	1.01, 0.5	1.00, 0.4	0.15	1.00, 0.5	1.01, 0.5	0.06
Serum Albumin, in gm/dl	4.23, 0.4	4.2, 0.4	0.056	4.2, 0.4	4.2, 0.4	0.92
Hematocrit, %	41.9, 4.5	42, 4.4	0.61	42.1, 4.4	41.9, 4.6	0.055
Platelet count, in thousands	250.6, 73.2	251.7, 72.4	0.85	251, 71.9	253.3, 71.4	0.9
DM, %	1392, 24.3%	680, 21.3%	<0.001	731, 23%	675, 21.2%	0.03
Smoker, %	859, 15%	433, 13.6%	0.072	464, 14.6%	430, 13.5%	0.58
COPD, %	229, 4%	107, 3.3%	0.145	117, 3.7%	105, 3.3%	0.20
Heart failure, %	183, 3.2%	82, 2.6%	0.079	98, 3.1%	60, 1.9%	<0.001
HTN, %	3542, 61.8%	1918, 60%	0.098	1968, 61.9%	1933, 60.7%	0.13
Preop dialysis,%	10, 0.2%	6, 0.2%	0.886	10, 0.3%	9, 0.3%	0.74
ASA ¾, %	3737, 65.2%	1965, 61.5%	<0.001	1911, 60.1%	2366, 60.3%	0.10

**Table 2:** 30-day complications across outpatient and inpatient RPN cohorts, after propensity matching SSI: surgical site infection, DVT: deep venous thrombosis, UTI: urinary tract infection, OR: operating room

	After propensity matching		
	Inpatient (n = 3,180)	Outpatient (n = 3,185)	p-value
Superficial SSI	0	0	*
Deep SSI	2, 0.06%	3, 0.09%	0.929
Organ Space SSI	25, 0.8%	17, 0.5%	0.227
Wound disruption	3, 0.09%	4, 0.1%	0.402
Pneumonia	28, 0.9%	14, 0.4%	0.009
DVT	16, 0.5%	6, 0.2%	0.044
Pulmonary Embolism	16, 0.5%	7, 0.2%	0.032
UTI	51, 1.6%	39, 1.2%	0.132
Stroke	6, 0.2%	2, 0.1%	0.223
Myocardial Infarction	9, 0.3%	2, 0.1%	0.029
Blood transfusion	76, 2.4%	16, 0.5%	<0.001
Sepsis	16, 0.5%	11, 0.3%	0.322
Septic shock	6, 0.2%	1, 0.01%	0.047
Return to OR/ Reoperation	48, 1.5%	24, 0.8%	0.001
Unplanned readmission	156, 4.9%	118, 3.7%	0.009
Unplanned readmission related to the procedure	118, 3.7%	90, 2.8%	0.095

Logistic Regression Analysis

A multivariate logistic regression analysis was then performed to analyze if the clinical setting was associated with increased odds of infectious complications (SSI, UTI, pneumonia, sepsis, and septic shock), reoperations, and readmissions. For infectious complications, significant factors included dependent functional status (OR: 2.51), stent placement during surgery (OR: 1.46), chronic obstructive pulmonary disease (OR: 1.39), inpatient setting (OR: 1.31), ASA classification 3-4 (OR: 1.29), BMI > 30 kg/m² (OR: 1.28), operation time > 180 minutes (OR: 1.26), and diabetes mellitus (OR: 1.25).

For reoperation, significant associations were observed with congestive heart failure (OR: 2.00), inpatient setting (OR: 1.92), stent placement (OR: 1.72), smoking (OR: 1.51), age > 75 years (OR: 1.50), ASA classification 3-4 (OR: 1.40), and operation time > 180 minutes (OR: 1.36).

For readmission, significant factors included congestive heart failure (OR: 1.91), ASA classification 3-4 (OR: 1.42), stent placement (OR: 1.42), operation time > 180 minutes (OR: 1.40), age > 75 years (OR: 1.37), and inpatient setting (OR: 1.23) (Table 3).

**Table 3:** Multivariate logistic regression analysis for 30-day infectious complications, reoperation, and readmissions after MIPN. OR: odds ratio, CI: confidence interval

Factors	Infectious complications		Reoperation		Readmission	
	OR, 95% CI	p-value	OR, 95% CI	p-value	OR, 95% CI	p-value
Age > 75 years	1.21 (0.90–1.54)	0.07	1.50 (1.07–2.11)	0.02	1.37 (1.13–1.66)	<0.001
BMI > 30 kg/m^2^	1.28 (1.11–1.48)	<0.001	0.94 (0.74–1.18)	0.58	0.96 (0.84–1.10)	0.55
Inpatient clinical setting, as compared to an outpatient setting	1.31 (1.12–1.53)	<0.001	1.92 (1.43–2.59)	<0.001	1.23 (1.06–1.42)	0.01
Diabetes mellitus	1.25 (1.07–1.46)	0.01	1.19 (0.91–1.55)	0.20	1.15 (0.99–1.33)	0.07
Hypertension	0.93 (0.80–1.08)	0.36	0.96 (0.75–1.24)	0.75	1.05 (0.91–1.21)	0.51
Smoker	1.03 (0.86–1.24)	0.76	1.51 (1.14–2.00)	<0.001	0.98 (0.81–1.17)	0.78
Chronic obstructive pulmonary disease	1.39 (1.01–1.91)	0.04	1.26 (0.76–2.11)	0.37	1.13 (0.82–1.56)	0.44
Congestive heart failure	1.17 (0.76–1.81)	0.48	2.00 (1.12–3.57)	0.02	1.91 (1.36–2.69)	<0.001
Chronic kidney disease requiring dialysis	1.42 (0.90-2.04)	0.42	0.81 (0.11–5.88)	0.83	1.22 (0.49–3.06)	0.67
Dependent functional status	2.51 (1.63–3.87)	<0.001	0.76 (0.24–2.38)	0.63	1.57 (0.97–2.53)	0.07
American Society of Anesthesiologists Classification System 3/4/5	1.29 (1.11–1.51)	<0.001	1.40 (1.07–1.82)	0.01	1.42 (1.23–1.65)	<0.001
Stent placement during surgery	1.46 (1.06–2.01)	0.02	1.72 (1.06–2.81)	0.03	1.42 (1.05–1.94)	0.02
Operation time > 180 minutes	1.26 (1.10–1.44)	0.001	1.36 (1.08–1.71)	0.01	1.40 (1.23–1.59)	<0.001

## Discussion

This study presents a comprehensive national analysis of RPN performed in outpatient versus inpatient settings, using data from the ACS-NSQIP. Our findings demonstrate a significant and steady increase in the proportion of MIPNs performed on an outpatient basis between 2019 and 2023, rising from 20.8% to 35.5%. This trend reflects a broader shift in surgical practice toward ambulatory care, even for complex oncologic procedures [11-12]. Importantly, after rigorous propensity score matching to control for demographic, clinical, and operative variables, outpatient RPN was associated with lower rates of several postoperative complications, including pneumonia, pulmonary embolism, myocardial infarction, deep vein thrombosis, blood transfusions, septic shock, reoperation, and overall unplanned readmissions. However, these findings should not be interpreted as evidence that outpatient surgery is inherently safer or superior. Rather, they support the conclusion that outpatient RPN is non-inferior to inpatient RPN in appropriately selected patients.

The increasing adoption of outpatient surgery in urology mirrors broader trends across surgical disciplines, driven by advances in minimally invasive techniques, enhanced recovery protocols, and institutional efforts to reduce healthcare costs. In urology, outpatient procedures have become standard for many benign conditions, and there is growing interest in extending this model to oncologic surgeries [11-14]. Our study confirms that this transition is well underway in the context of partial nephrectomy, a technically demanding procedure traditionally associated with inpatient care. The observed increase in outpatient MIPN likely reflects growing surgeon confidence, improved perioperative management, and evolving institutional policies that support same-day discharge for selected patients [15,16].

Despite the use of propensity score matching to minimize selection bias, residual differences persisted between the inpatient and outpatient cohorts. Notably, the prevalence of diabetes remained significantly higher in the inpatient group, even after matching. After matching, the prevalence of CHF in the inpatient cohort became significantly higher than the prevalence in the outpatient cohort; this observation could be attributed to a statistical phenomenon called propensity matching paradox, where imbalances in the distribution of covariates become exaggerated. Drawing conclusions based on this matching should be inferred with caution [17]. Understanding this caveat, the results of matching still suggest that patients with more complex medical profiles continue to be preferentially selected for inpatient care [18,19]. Diabetes and CHF are well-established risk factors for perioperative complications, and their higher prevalence in the inpatient cohort may partially explain the increased rates of adverse outcomes observed in this group. These findings underscore the importance of careful patient selection in determining surgical settings and highlight the limitations of statistical matching in fully accounting for clinical complexity.

The observed differences in outcomes between outpatient and inpatient RPN must be interpreted with caution. While outpatient surgery was associated with lower complication rates, this likely reflects the selection of healthier patients for ambulatory care rather than an intrinsic advantage of the outpatient setting. Indeed, the safety of outpatient RPN is contingent upon appropriate patient selection, institutional readiness, and adherence to structured perioperative protocols. Our findings support the growing consensus that outpatient RPN can be safely performed in selected patients without compromising short-term outcomes, but they do not suggest that all, or even a majority of patients, are suitable candidates for same-day discharge.

An important and often overlooked aspect of outpatient surgical care is the potential for disparities in access. In our study, a significantly higher proportion of White patients underwent outpatient RPN compared to other racial and ethnic groups. This disparity persisted even after propensity matching and raises important questions about equity in surgical care delivery. The reasons for this difference are likely multifactorial and may include differences in socioeconomic status, insurance coverage, geographic access to high-volume centers, implicit bias in clinical decision-making, and patient preferences [20-22]. Regardless of the underlying causes, the observed disparity highlights the need for further investigation into the structural and systemic barriers that may limit access to outpatient surgical care for underrepresented populations. Ensuring equitable access to high-quality, cost-effective surgical options must be a priority as outpatient surgery continues to expand [23].

The economic implications of outpatient RPN are also significant. Renal cell carcinoma (RCC) is associated with substantial healthcare costs, and the financial burden is expected to increase with rising incidence and longer survival [3,4]. Outpatient surgery offers a cost-effective alternative to traditional inpatient care by reducing hospital length of stay, minimizing resource utilization, and selecting patients appropriately [24]. Prior studies have demonstrated that outpatient procedures can reduce total episode-of-care costs by 20-40%, and our findings support the notion that outpatient RPN may deliver high-value care without compromising patient safety. In addition to cost savings, outpatient surgery offers several patient-centered benefits, including improved satisfaction, faster return to normal activities, and reduced risk of hospital-acquired complications. These advantages are particularly relevant in the post-COVID era, where minimizing hospital exposure has become a priority for both patients and providers [25-27].

This study has several strengths that enhance the validity and generalizability of its findings. The use of the ACS-NSQIP database provides access to a large, diverse, and nationally representative sample of patients undergoing RPN across a wide range of institutions. This enhances the external validity of our results and allows for the identification of real-world trends in surgical practice. The application of rigorous propensity score matching using a comprehensive set of demographics, clinical, and operative variables minimizes selection bias and strengthens the internal validity of our comparisons. The inclusion of a wide array of postoperative outcomes, including infectious, cardiovascular, and procedural complications, provides a holistic assessment of surgical safety. Finally, the temporal analysis of outpatient surgery trends adds a valuable dimension to the study, highlighting the dynamic evolution of surgical practice over time.

Nonetheless, several limitations must be acknowledged. One of the limitations is the inability to distinguish between laparoscopic and robotic approaches within the MIPN cohort while analyzing the trends from 2019 to 2023. In addition, the NSQIP database does not capture detailed oncologic variables such as tumor size, location, complexity (e.g., RENAL nephrometry score), or pathologic staging. These factors are critical in determining the technical difficulty of partial nephrectomy and may influence both the decision to pursue outpatient surgery and the risk of postoperative complications. The absence of these data limits our ability to fully adjust for tumor-related complexity and may introduce residual confounding. Another limitation is the reliance on administrative definitions of inpatient and outpatient status, which may vary across institutions and may not always reflect true clinical practice. Although we corroborated these classifications using length of stay data, misclassification remains a possibility. Furthermore, while the NSQIP database captures 30-day postoperative outcomes, it does not provide information on long-term oncologic outcomes, renal function, or patient-reported quality of life. These endpoints are essential for a comprehensive evaluation of the safety and efficacy of outpatient RPN and should be the focus of future research.

The findings of this study have several important implications for clinical practice and healthcare policy. They support the continued expansion of outpatient surgical programs for appropriately selected patients undergoing RPN. Institutions should consider developing standardized protocols for outpatient partial nephrectomy, including preoperative risk stratification, enhanced recovery pathways, and structured postoperative follow-up. Our results also highlight the need for further research to refine patient selection criteria and identify predictors of successful outpatient surgery. Future studies should aim to capture long-term outcomes such as oncologic control and changes in renal function.

## Conclusions

In this large, national cohort study using ACS-NSQIP data, we found that outpatient MIPN has become increasingly common over recent years and that outpatient RPN is associated with favorable short-term outcomes when compared to inpatient cases. However, these findings should be interpreted in the context of careful patient selection, as outpatient surgery is not inherently superior but rather non-inferior in appropriately selected individuals. The persistence of disparities, particularly the higher proportion of White patients undergoing outpatient surgery, highlights the need for equitable access to ambulatory surgical care. While outpatient RPN offers potential benefits in terms of safety, cost-effectiveness, and patient satisfaction, its success depends on careful patient selection, institutional readiness, and structured perioperative protocols.

## References

[1] Kocarnik JM, Compton K, Dean FE (2022). Cancer incidence, mortality, years of life lost, years lived with disability, and disability-adjusted life years for 29 cancer groups from 2010 to 2019: a systematic analysis for the Global Burden of Disease Study 2019. JAMA Oncol.

[2] Chen R, Tang T, Han J (2025). Temporal trends of the disease burden of renal cell carcinoma from 1992 to 2019 in the US: a population-based analysis. Cancer Causes Control.

[3] Bhandari NR, Kale HP, Carroll NV (2022). Healthcare costs and resource utilization associated with renal cell carcinoma among older Americans: a longitudinal case-control study using the SEER-Medicare data. Urol Oncol.

[4] Kale HP, Mays DP, Nadpara PA, Slattum PW, Paul AK, Carroll NV (2019). Economic burden of renal cell carcinoma among older adults in the targeted therapy era. Urol Oncol.

[5] Chen YW, Wang L, Panian J (2023). Treatment landscape of renal cell carcinoma. Curr Treat Options Oncol.

[6] Leslie S, Goh AC, Gill IS (2013). Partial nephrectomy--contemporary indications, techniques and outcomes. Nat Rev Urol.

[7] Leow JJ, Heah NH, Chang SL, Chong YL, Png KS (2016). Outcomes of robotic versus laparoscopic partial nephrectomy: an updated meta-analysis of 4,919 patients. J Urol.

[8] Pellegrino A, Briganti A, Crivellaro S (2025). Same-day outpatient robotic surgery in urology. Eur Urol Focus.

[9] Madhusoodanan V, Carto C, Parmar M, Ritch C, Parekh DJ, Ramasamy R (2022). Trends in outpatient versus inpatient urologic surgery at a university academic medical center. Curr Opin Urol.

[10] Johnson K, Lane BR, Weizer AZ (2021). Partial nephrectomy should be classified as an inpatient procedure: results from a statewide quality improvement collaborative. Urol Oncol.

[11] Young S, Osman B, Shapiro FE (2023). Safety considerations with the current ambulatory trends: more complicated procedures and more complicated patients. Korean J Anesthesiol.

[12] Bal DS, Chung D, Dhillon H (2024). The safety and efficacy of ambulatory urologic surgery A paradigm shift towards optimizing resource use in outpatient settings. Can Urol Assoc J.

[13] Soputro NA, Ramos-Carpinteyro R, Chavali JS, Pedraza AM, Mikesell CD, Kaouk J (2025). Predictors for selection of outpatient single-port robot-assisted laparoscopic radical prostatectomy. BJU Int.

[14] Abaza R, Murphy C, Bsatee A, Brown DH Jr, Martinez O (2021). Single-port robotic surgery allows same-day discharge in majority of cases. Urology.

[15] Siron N, Assad A, Ouirzanne M (2023). Performing urological inpatient procedures as same-day procedures during the COVID pandemic A retrospective feasibility study. Can Urol Assoc J.

[16] Ploussard G, Almeras C, Beauval JB, Gautier JR, Loison G, Salin A, Tollon C (2022). Same-day discharge surgery for robot-assisted radical prostatectomy in the era of ERAS and prehabilitation pathways: a contemporary, comparative, feasibility study. World J Urol.

[17] Ripollone JE, Huybrechts KF, Rothman KJ, Ferguson RE, Franklin JM (2018). Implications of the propensity score matching paradox in pharmacoepidemiology. Am J Epidemiol.

[18] Rajan N, Rosero EB, Joshi GP (2021). Patient selection for adult ambulatory surgery: a narrative review. Anesth Analg.

[19] Kulkarni S, Harsoor SS, Chandrasekar M (2017). Consensus statement on anaesthesia for day care surgeries. Indian J Anaesth.

[20] Tsai TC, Brownlee SA, Dai D (2025). Disparities in access to outpatient surgery related to removal of procedures from Medicare's inpatient-only list. Ann Surg.

[21] Amba V, Izadi S, Ramesh T, Yu H (2025). Outpatient surgical institutions in the rural United States: trends from 2010 to 2020. Am J Surg.

[22] Janeway MG, Sanchez SE, Chen Q, Nofal MR, Wang N, Rosen A, Dechert TA (2020). Association of race, health insurance status, and household income with location and outcomes of ambulatory surgery among adult patients in 2 US states. JAMA Surg.

[23] Chatterjee A, Amen TB, Khormaee S (2022). Trends in geographic disparities in access to ambulatory surgery centers in New York, 2010 to 2018. JAMA Health Forum.

[24] Kaye DR, Luckenbaugh AN, Oerline M, Hollenbeck BK, Herrel LA, Dimick JB, Hollingsworth JM (2020). Understanding the costs associated with surgical care delivery in the Medicare population. Ann Surg.

[25] Patel HD, Matlaga BR, Ziemba JB (2019). Trends in the setting and cost of ambulatory urological surgery: an analysis of 5 states in the healthcare cost and utilization project. Urol Pract.

[26] Nguyen DD, Marchese M, Ozambela M, Bhojani N, Ortega G, Trinh QD, Friedlander DF (2020). Ambulatory-based bladder outlet procedures offer significant cost savings and comparable 30-day outcomes relative to inpatient procedures. J Endourol.

[27] Berger A, Friedlander DF, Herzog P, Ortega G, O'Leary M, Kathrins M, Trinh QD (2019). Impact of index surgical care setting on perioperative outcomes and cost following penile prosthesis surgery. J Sex Med.

